# How adverse childhood experiences relate to single and multiple health risk behaviours in German public university students: a cross-sectional analysis

**DOI:** 10.1186/s12889-018-5926-3

**Published:** 2018-08-13

**Authors:** Jascha Wiehn, Claudia Hornberg, Florian Fischer

**Affiliations:** 1 Department of Public Health Medicine, School of Public Health, Bielefeld University, P.O. Box 100 131, 33501 Bielefeld, Germany; 2 Department of Environment and Health, School of Public Health, Bielefeld University, P.O. Box 100 131, 33501 Bielefeld, Germany

**Keywords:** Adverse childhood experiences, Child maltreatment, Violence, Risky behaviours, Stress-related trauma, Health, Life course approach, Germany

## Abstract

**Background:**

Adverse childhood experiences (ACEs) have been shown to be linked to health risk behaviours (HRBs). This study aims to identify risk factors for ACEs and to examine the associations between ACEs and single and multiple HRBs in a sample of university students in Germany.

**Methods:**

An online-based cross-sectional study was conducted among public university students (*N* = 1466). The widely applied ACE questionnaire was used and extended to operationalise 13 categories of childhood adversity. First, variables for each type of ACE and HRB were dichotomised (single ACEs and single HRBs), and then used for cumulative scores (multiple ACEs and multiple HRBs). Frequencies were assessed, and (multinomial) logistic regression analyses were performed.

**Results:**

Prevalence rates of ACEs ranged from 3.9 to 34.0%, depending on the type of childhood adversity. Sociodemographic risk and protective factors for single/multiple ACEs varied strongly depending on the outcome. In particular, a high family socioeconomic status seemed to be a consistent protective factor for most ACEs. After adjusting for sociodemographic characteristics, both single and multiple HRBs were associated with single events of ACEs. Moreover, dose-response relationships between multiple ACEs and various single and multiple HRBs were found.

**Conclusions:**

The study provides strong evidence that ACEs are associated with HRBs. The number of ACEs may play a role in single or multiple HRBs. Reducing the number of ACEs could thus decrease HRBs, which account for many of the leading causes of morbidity and death. The findings highlight the importance of trauma-informed health interventions designed to prevent the occurrence of ACEs, and build capacity among children and adults.

**Electronic supplementary material:**

The online version of this article (10.1186/s12889-018-5926-3) contains supplementary material, which is available to authorized users.

## Background

Adverse childhood experiences (ACEs) are traumatic and stress-related events during childhood, such as sexual abuse or growing up with a substance-abusive household member. ACEs not only cause immediate health hazards, but also affect health across the lifespan [1].

Strong empirical evidence exists suggesting that exposure to high numbers of ACEs increases the risk of many of the leading causes of death [2] and can reduce life expectancy by almost 20 years [3]. ACEs can cause direct health consequences, such as somatic and psychosomatic disorders, cognitive-emotional reactions [4] or even death [5]. Moreover, ACEs can also have a negative impact throughout the entire life course by affecting the individual’s physical health [6–8] and mental health [9–11]. Moreover, it has been found that experiencing trauma during childhood can be linked to various health risk behaviours (HRBs) later in life [12]. Overall, there seems to be strong evidence to indicate that exposure to ACEs can lead to risky alcohol consumption [13–15], smoking [6, 15, 16], illicit drug use [15, 17, 18], sexual risk behaviours [19–21], and suicidal behaviour [21, 22]. Since exposure to ACEs is rarely found to occur in isolation [23, 24], it is essential to understand that children experiencing multiple types of maltreatment have a greater vulnerability [25].

The occurrence of ACEs is the product of a complex interplay between multiple risk and protective factors. Following an ecological perspective [26], these key factors can appear at an individual level, in social relationships, in a community context, and in a societal layer increasing the risk of ACEs [27]. In addition to these adverse effects, protective factors (e.g., support from a trusted adult) can mitigate the risk of ACEs [28].

Since the initial ACE study in 1998 [1], health professionals around the world have started exploring the prevalence of ACEs. Stoltenborgh et al. [29] estimated that 22.6% of children and adolescents around the world were physically abused, and 36.3% emotionally abused, 16.3% were physically neglected, 18.4% were emotionally neglected, and 18% of the females – compared to 13.4% of the males – reported that they had experienced sexual abuse. In Europe, the prevalence of physical abuse during childhood is thought to be as high as 22.9%, with a prevalence of emotional abuse of 29.1%, while 13.4% of female and 5.7% of male Europeans report having experienced sexual abuse [30]. Bellis et al. [31] reported overall prevalence rates from eight Eastern European countries for physical abuse (18.6%), alcoholism in a member of the household (16.4%), domestic violence towards the mother (14.6%), parental separation or divorce (14.1%), emotional neglect (11.8%), a depressed or suicidal member of the household (10.0%), emotional abuse (8.0%), sexual abuse (7.5%), an incarcerated household member (5.3%), and drug abuse by a member of the household (2.6%).

Although extensive research has been conducted in Germany to investigate the prevalence rates and potentially harmful effects of specific subtypes of ACEs (e.g., physical abuse), more research is needed on the broader concept of ACEs and their influence. No single study was found which assessed the prevalence rates of self-reported ACEs in a German non-patient sample. In fact, previous studies in Germany used clinical samples [32–34]. However, a few studies estimating self-reported prevalence rates of child maltreatment within the German population exist [35–40]. But, compared to ACE studies, such child maltreatment studies only cover a few categories of the ACE concept (physical/emotional/sexual abuse and physical/emotional neglect) [41], and therefore do not account for other forms of ACE – such as growing up with a depressive household member or being bullied. According to these investigations on child maltreatment, it can be assumed that prevalence rates range from 4.4% [36] to 74.9% [40] for physical abuse, 3.5% [36] to 11.9% [35] for emotional abuse, 14.6% [36] to 48.4% [37] for physical neglect, 11.8% [36] to 40.7% [35] for emotional neglect, and 3.2% [36] to 12.8% [40] for sexual abuse.

This study was conducted in order to close this research gap by estimating prevalence rates for ACEs and determining associations between sociodemographic characteristics and ACEs as well as ACEs and HRBs among public university students in Germany. The research questions were:

1. How high are the prevalence rates for ACEs during the first 18 years of life among public university students in Germany?
2. How are sociodemographic factors and single/multiple ACEs associated?
3. How are single/multiple ACEs and single/multiple HRBs associated after controlling for sociodemographic factors?

## Methods

### Study design and participants

A cross-sectional study among public university students was conducted across German public universities from May to June 2017 using the web-based survey tool “EFS Survey”. From a list of all 728 faculties located in public universities in Germany, 176 were randomly chosen and requested to participate. All faculties had an equal chance of being included in the sample. This procedure was used to reduce the risk of sampling bias. Unfortunately, 80 faculties (45.5%) did not reply at all, 72 (40.9%) refused to participate (no mailing list in existence, no access to mailing list, no interest in research, overload of requests, etc.), and only 24 (13.6%) agreed to support the study. Then, the participating faculties distributed the invitations to the online survey to their students via mailing lists. Where a mailing list was not available, other media channels were used to distribute the link to the students of the respective faculty (e.g., closed Facebook groups). Students were informed about the context, the methodology, the possibility to withdraw participation with no adverse consequences, the anonymous and voluntary nature of the study, as well as the potential risks of emotional distress. Participants gave their informed consent by clicking on a button before proceeding with the survey. Upon completion, participants were provided with related links and could therefore retrieve further information or seek help if necessary. The study was approved by the Ethics Committee of Bielefeld University.

The link to the online questionnaire was opened 2496 times. Since many closed the online questionnaire immediately after opening the first page, the initial data set consisted of 1833 cases. In order to include only those cases that provided sufficient information for further analysis, participants with 15 or more missing values on ACE variables (*n* = 298), and participants with 7 or more missing values on HRBs items (*n* = 34), were excluded from the sample. For the purpose of reliable gender-related analyses, cases where sex was not indicated (*n* = 16), or where the respondents indicated a gender other than male or female (*n* = 8), were eliminated from the dataset. Finally, to ensure that only students were in the sample, participants who did not answer the item about their age and participants older than 29 who did not indicate their university of study were excluded (*n* = 11). The net sample consisted of 1466 university students.

Unfortunately, response rates could not be calculated due to a lack of information about the total number of students who had received the invitation. Compared to all German students enrolled at public universities [42], females were overrepresented in this sample (80.3%). The mean age of participants was 24.1 years (SD = 4.5), and the respondents’ parents were slightly older than the mean age for the entire German population [43]. Most respondents reported having a non-immigrant background (84.8%), which is more than that estimated for the overall German population [44]. Two thirds of the participants (66.6%) answered that at least one of their parents had a university degree. According to respondents, their families’ socio-economic status (SES) increased continuously during their childhood. In total, the SES of the sample was 1.98 (SD = 0.8) over all three time points. Table 1 shows the demographic characteristics of the sample.

**Table 1:** Sociodemographic characteristics, prevalence rates of adverse childhood experiences (ACEs) and health risk behaviours (HRBs) of a sample of 1466 university students

Characteristics	Total (*N* = 1466)		Females (*n* = 1183)		Males (*n* = 283)		
	*n* ^a^	%^b^	*n* ^a^	%^b^	*n* ^a^	%^b^	*p* ^c^
Individual age (*mean*, SD)	24.09 (4.49)	–	24.03 (4.51)	–	24.37 (4.42)	–	0.309
Parental age (*mean*, SD)	55.31 (6.26)	–	55.25 (6.27)	–	55.56 (6.22)	–	0.846
Migration background							0.025
Non-migration background	1240	84.8	1010	86.9	230	82.7	
Migration background	199	15.2	151	13.1	48	17.3	
Parental education							0.630
Non-college degree	478	33.4	383	32.8	95	34.0	
College degree	972	66.6	787	67.2	185	66.0	
Family socioeconomic status							
5 years (*mean*, SD)	1.60 (1.01)	–	1.59 (0.10)	–	1.63 (1.04)	–	0.252
10 years (*mean*, SD)	2.04 (0.94)	–	2.05 (0.93)	–	2.01 (0.96)	–	0.594
15 years (*mean*, SD)	2.30 (0.83)	–	2.30 (0.83)	–	2.28 (0.84)	–	0.583
Total (*mean*, SD)	1.98 (0.80)	–	1.98 (0.79)	–	1.97 (0.80)	–	0.457
ACEs							
Physical abuse	62	3.9	53	4.6	9	3.2	0.189
Emotional abuse	300	19.6	251	21.7	49	17.7	0.060
Physical neglect	64	4.6	50	4.3	14	5.0	0.531
Emotional neglect	266	19.1	209	17.8	57	20.4	0.200
Sexual abuse	215	12.3	191	16.4	24	8.5	< 0.001
Substance abuse by a household member	184	12.5	150	12.9	34	12.2	0.719
Mental illness of a household member	453	32.1	366	32.1	87	32.2	0.967
Domestic violence	473	34.0	375	32.5	98	35.4	0.248
Parental separation/divorce	408	28.1	329	27.9	79	28.3	0.866
Absence/ death of caregiver	262	18.5	208	17.7	54	19.3	0.426
Financial problems	150	9.8	125	10.7	25	8.0	0.273
Bullying	251	20.8	178	15.2	73	26.0	< 0.001
Peer Violence	49	5.5	25	2.1	24	8.6	< 0.001
Multiple ACEs							0.387
None	412	27.9	334	28.2	78	27.5	
1 ACE	348	22.9	287	24.3	61	21.5	
2 ACEs	216	15.7	167	14.1	49	17.3	
3 ACEs	135	9.0	111	9.4	24	8.5	
4+ ACEs	355	24.6	284	24.0	71	25.1	
HRBs							
Risky drinking	299	18.9	252	21.3	47	16.6	0.023
Smoking daily	153	11.2	118	10.0	35	12.4	0.160
Drug abuse	206	17.9	141	12.2	65	23.4	< 0.001
Early sexual intercourse	279	17.5	240	21.0	39	14.3	0.001
Multiple sexual partners	63	5.3	49	4.7	14	5.8	0.376
Suicidality	138	11.4	101	8.9	37	13.8	0.004
Multiple HRBs							0.200
None	755	49.7	622	52.6	133	47.0	
1 HRB	418	29.7	329	27.8	89	31.5	
2 HRBs	190	13.4	150	12.7	40	14.1	
3+ HRBs	103	7.2	82	6.9	21	7.4	

Note: Parental age: The parents’ age was calculated by averaging the age of both parents; migration background: non-migration background = both parents born in Germany, migration background = one or both parents born outside of German; parental education: non-college degree = neither parent has a college degree, college degree = one or both parents with college degree; family’s socioeconomic status (SES): retrospective, self-reported socioeconomic status of the family according to household possessions at the age of 5 years, 10 years, and 15 years

^a^Results on absolute frequencies (*n*) are unweighted

^b^Results on relative frequencies (%) are weighted

^c^*p*-value (two-sided) based on Pearson’s χ^2^ test

### Measures

#### Sociodemographic and economic factors

Participants were asked about their sex, age, parents’ birthplaces, and parents’ level of education. Age of parents was calculated by averaging the age of both parents. The household’s SES during the participant’s childhood was assessed using the SC Childhood Interview measure [45]. Participants completed questions relating to three potential wealth indicators. The method described by John-Henderson et al. [46] was modified so that each indicator was measured for the ages of 5, 10 and 15 years. A sum score was calculated for each point in time (range 0–3). These scores were then averaged into a total score across all three points in time during childhood.

#### Adverse childhood experiences

Thirteen types of ACEs were assessed by adopting the Adverse Childhood Experiences International Questionnaire (ACE-IQ) [47]. Two additional ACE categories (serious financial problems; caregiver’s absence) were taken from an extended and validated ACE-IQ [48]. It should be noted that, in order to avoid inferential statistical analysis using low variances with predictor variables, physical fighting and violence in the community were combined (=peer violence) and death of a caregiver was pooled with parental absence (=absence/death of a caregiver). As can be seen in Table 1, this resulted in a total number of 13 ACEs. As an example of how ACEs were operationalised, physical abuse was assessed by asking participants two questions:

1. *During the first 18 years of your life how often did a parent, guardian or household member punch, kick or beat you?* (*never*; *once*; *a few times*; *many times*).
2. *During the first 18 years of your life how often did a parent, guardian or household member hit you with an object?* (*never*; *once*; *a few times*; *many times*).

The Appendix shows the wording (translated from the German), response scales and responses to all items assessing ACEs (see Additional file 1). In order to decrease socially desirable response behaviour, participants were allowed to skip sensitive questions. Those who indicated that they could not or did not want to give an answer were treated as missing values. Variables were dichotomised according to the coding manual for the frequency version provided by the World Health Organisation (WHO) [47]. As a result, a person was regarded as being exposed to a given traumatic event during childhood if at least one question relating to each type of ACE was marked as positive. Moreover, a cumulative ACE score was calculated by summing all ACEs (range: 0–13).

#### Health risk behaviours

Consumption of alcohol was measured using the brief version of the Alcohol Use Disorders Identification Test (AUDIT-C) [49]. The AUDIT-C includes three questions on drinking habits using ordinal response options. As suggested by the Robert Koch Institute (RKI) [50], a sum score was calculated using these three questions, where each scored 0–4 points (total range 0–12). Following recommendations by DeMartini and Carey [51], relatively high cut-off points were selected to divide the sample into risky or non-risky college drinkers (females: ≥5 points; males: ≥7 points). In order to quantify smoking habits, a single item was taken from the DEGS study [52]: *Do you currently smoke – even just occasionally?* (*yes, every day*; *yes, occasionally*; *no, not anymore*; *never smoked*). Empirical evidence suggests that even occasional smoking can be clearly harmful [53]. However, tests in the statistical analyses revealed that a fairly stringent binary coding (daily smokers vs. occasional/former/non-smokers) resulted in the most robust models and was therefore chosen. Similar to the question on smoking habits [52], the question on drug abuse was: *Do you currently take illegal drugs – even just occasionally?* Due to the generally lower prevalence rates of drug vs. tobacco use [54, 55], participants were dichotomised as daily or occasional drug users vs. former drug users and non-users. Data on the participants’ early sexual behaviour was obtained by asking: *How old were you when you first had consenting sexual intercourse?* (*younger than 13 years*; *13 years*; *14 years*; *15 years*; *16 years*; *17 years*; *18 years or older*; and *no sexual intercourse yet*). A binary coding was performed (< 16 years vs. ≥16 years or more, or no sexual intercourse). In order to assess risky sexual behaviour, participants were asked how many sexual partners they had had over the past 12 months. A frequently used cut-off of four or more sexual partners vs. fewer than four was applied to dichotomise the variable [56–58]. Considering suicide during the past 12 months was used as a proxy for suicidal behaviour (*During the past 12 months, did you ever consider attempting suicide?*) [59]. Again, a cumulative score for HRBs was calculated by summing up all HRBs (range: 0–6).

### Pre-test

Between January and March 2017, the initial questionnaire was evaluated by first conducting cognitive interviews (*n* = 8), and then performing a quantitative pre-test among 146 university students from the School of Public Health, Bielefeld University. Changes (e.g., providing a definition of humiliation for the items on emotional abuse) were made as suggested by the participants. Since the second phase only brought up minor modifications to the questionnaire, it was possible to pool the pre-test and the main study sample to increase the power of the net sample.

### Data analysis

Data management and analysis were carried out using IBM SPSS Statistics 22. A post-stratification weighting factor, taking into account the unbalanced gender ratio (unweighted: 80.7% females; 19.3% males; weighted: 48.5% females; 51.5% males), was calculated using data retrieved from the Federal Statistical Office [42]. The weighting variable was applied in descriptive, bivariate, and multivariate analysis. Non-weighted results are only displayed for absolute numbers. Frequency runs for the total sample and stratified by gender were explored to gather descriptive data on sociodemographic characteristics and prevalence rates among the participants. Significance levels were set at the 5% alpha level using two-sided t-tests for independent samples for continuous variables, and two-sided Pearson’s Chi-square tests of independence for categorical variables. Before exploring potential associations between the variables of interest, statistical models were tested for multicollinearity. The variance inflation factor (VIF) revealed no evidence of multicollinearity (VIF < 2.00). Next, (multinomial) logistic regression analyses were conducted to identify the degree of association between the independent and the binary (or ordinal) dependent variables.

## Results

### Prevalence rates

Altogether, 73.8% of the participants were positively marked for any of the 13 ACEs. As presented in Table 1, ACE prevalence rates ranged from 3.9% (physical abuse) to 34.0% (exposure to domestic violence). The cumulative ACE score shows that 22.9% (*n* = 348) of the students had experienced one ACE, 15.7% (*n* = 216) two ACEs, 9.0% (*n* = 135) three ACEs, and 24.6% (*n* = 355) four or more ACEs. Significant gender-related differences (*p* < 0.001) were found for sexual abuse (females 16.4%, males 8.5%), bullying (females: 15.2%, males: 26.0%), and peer violence (females 2.1%, males 8.6%).

### Risk factors for adverse childhood experiences

The results in Table 2 show a range of 2.6–17.4% for explained variances of the statistical models. All predictor variables had a significant influence in at least one model. However, some variables were more relevant for explaining and predicting ACEs than others; for example, a higher SES had a significant (*p* < 0.05) protective effect against the occurrence of 12 of the 13 ACEs, while age was found to have a small but significant impact on the occurrence of emotional neglect and parental separation or divorce. Significant ORs showed a range across the 13 dependent variables of 0.37 (95% CI, 0.29–0.47) to 3.91 (95% CI, 2.16–7.06).

**Table 2:** Logistic regression analyses on associations between sociodemographic factors and single types of adverse childhood experiences (ACEs)

Sociodemographic characteristics	PA (*n* = 62)	EA (*n* = 300)	PN (*n* = 64)	EN (*n* = 266)	SA (*n* = 215)	SubA (*n* = 184)	MI (*n* = 453)	DV (*n* = 473)	SD (*n* = 408)	AD (*n* = 262)	FP (*n* = 150)	B (*n* = 251)	PV (*n* = 49)
	OR (95% CI)	OR (95% CI)	OR (95% CI)	OR (95% CI)	OR (95% CI)	OR (95% CI)	OR (95% CI)	OR (95% CI)	OR (95% CI)	OR (95% CI)	OR (95% CI)	OR (95% CI)	OR (95% CI)
Female (ref.: male)	1.65 (0.93–2.93)	**1.36 (1.03–1.79)**	0.86 (0.52–1.44)	0.88 (0.66–1.16)	**1.98 (1.42–2.77)**	1.05 (0.76–1.44)	0.97 (0.77–1.22)	0.92 (0.74–1.16)	1.02 (0.80–1.29)	0.92 (0.70–1.21)	1.27 (0.87–1.84)	**0.50 (0.38–0.65)**	**0.22 (0.12–0.41)**
Individual age^a^	1.01 (0.95–1.07)	1.00 (0.97–1.04)	0.99 (0.94–1.07)	**1.07 (1.03–1.11)**	0.96 (0.92–1.01)	1.01 (0.97–1.06)	1.01 (0.97–1.04)	1.03 (0.99–1.06)	**1.05 (1.01–1.08)**	1.03 (0.99–1.07)	1.02 (0.97–1.07)	1.02 (0.98–1.06)	1.0 (0.94–1.07)
Parental age^a^	1.04 (0.99–1.09)	**1.03 (1.0–1.06)**	**1.06 (1.0–1.11)**	0.99 (0.96–1.02)	**1.07 (1.04–1.11)**	**1.03 (1.0–1.07)**	1.02 (0.99–1.04)	1.01 (0.99–1.03)	**0.95 (0.93–0.98)**	1.01 (0.98–1.03)	0.97 (0.94–1.01)	**1.04 (1.01–1.06)**	**1.08 (1.04–1.13)**
Migration background (ref.: none)	**3.91 (2.16–7.06)**	**1.92 (1.37–2.70)**	0.62 (0.29–1.33)	**2.11 (1.50–2.97)**	0.92 (0.58–1.46)	1.11 (0.72–1.69)	1.19 (0.87–1.64)	**2.29 (1.69–3.12)**	**0.67 (0.47–0.95)**	1.13 (0.79–1.64)	1.46 (0.93–2.28)	1.0 (0.70–1.45)	1.20 (0.65–2.19)
Low parental education (ref.: high)	**2.06 (1.15–3.67)**	1.08 (0.81–1.45)	0.97 (0.56–1.67)	1.20 (0.90–1.61)	1.35 (0.96–1.91)	1.24 (0.88–1.73)	0.94 (0.73–1.20)	1.25 (0.98–1.60)	0.87 (0.67–1.13)	0.86 (0.64–1.16)	**1.89 (1.30–2.76)**	**1.42 (1.08–1.87)**	1.50 (0.92–2.47)
Family SES^a^	**0.56 (0.39–0.80)**	**0.71 (0.60–0.85)**	**0.59 (0.43–0.82)**	**0.76 (0.63–0.91)**	**0.78 (0.63–0.97)**	**0.62 (0.50–0.77)**	**0.75 (0.64–0.87)**	**0.85** (0.73–0.99)	**0.57 (0.48–0.67)**	**0.64 (0.54–0.77)**	**0.37 (0.29–0.47)**	0.85 (0.71–1.01)	**0.67 (0.49–0.92)**
*n*	1403	1390	1396	1406	1405	1398	1367	1387	1411	1408	1406	1402	1397
R^2^	0.150	0.057	0.048	0.075	0.058	0.058	0.026	0.058	0.078	0.044	0.174	0.063	0.133

Note: Logistical regression, weighted results; significant values (*p* < 0.05) are in bold; *OR* odds ratio, *CI* confidence interval, *PA* physical abuse, *EA* emotional abuse, *PN* physical neglect, *EN* emotional neglect, *SA* sexual abuse, *SubA* substance abuse by household member, *MI* mental illness in the household, *SES* socioeconomic status, *DV* domestic violence, *SD* parental separation or divorce, *AD* parental absence or death, *FP* serious financial problems, *B* bullying, *PV* peer violence, *SES* socioeconomic status; parental age: the parents’ age was calculated by averaging the age of both parents; family SES: low-high family socioeconomic status during childhood

^a^Variables on individual age, parental age, and family SES were treated as metric variables in the regression models

Sociodemographic variables were then regressed on multiple ACEs (Table 3). In order to improve interpretability, continuous variables (individual/parental age; family SES) were categorised on the basis of their percentiles. Multinomial logistical regression analysis revealed that the model explained 18.8% of the variance. Again, family SES plays a key role in explaining and predicting the occurrence of ACEs. Students growing up in a household with a very low SES are 8.08 times likelier to be exposed to four or more ACEs than students growing up in a family with a very high SES (95% CI, 4.25–15.01).

**Table 3:** Multinominal logistic regression analyses on associations between sociodemographic factors and single types of adverse childhood experiences (ACEs)

Sociodemographic characteristics	1 ACE (*n* = 348)	2 ACEs (*n* = 216)	3 ACEs (*n* = 135)	4+ ACEs (*n* = 355)
	OR (95% CI)	OR (95% CI)	OR (95% CI)	OR (95% CI)
Female (ref.: male)	1.25 (0.86–1.81)	1.01 (0.67–1.53)	1.42 (0.85–2.35)	1.09 (0.76–1.58)
Individual age (ref.: > 25 years)				
< 21 years	1.05 (0.56–1.96)	1.08 (0.56–2.10)	**0.22 (0.08–0.59)**	0.69 (0.36–1.27)
21–22 years	0.73 (0.40–1.33)	0.56 (0.29–1.08)	**0.47 (0.23–0.97)**	**0.55 (0.31–0.99)**
23–25 years	1.01 (0.61–1.65)	0.64 (0.37–1.11)	**0.28 (0.14–0.56)**	**0.59 (0.35–0.92)**
Parental age (ref.: > 59 years)				
< 51 years	1.05 (0.55–2.01)	0.83 (0.41–1.66)	2.12 (0.81–5.56)	0.93 (0.51–1.71)
51–54 years	1.12 (0.65–1.93)	1.03 (0.59–1.83)	2.12 (0.95–4.75)	0.59 (0.35–1.00)
55–59 years	1.03 (0.61–1.76)	**0.54 (0.30–0.99)**	**2.77 (1.31–5.86)**	**0.49 (0.29–0.82)**
Migration background (ref.: none)	1.04 (0.60–1.80)	1.51 (0.87–2.64)	0.57 (0.25–1.33)	1.55 (0.94–2.58)
Low parental education (ref.: high)	1.12 (0.74–1.70)	1.32 (0.85–2.07)	1.70 (0.99–2.89)	**1.69 (1.13–2.51)**
Family SES (ref.: very high)				
Very low	**4.76 (2.49–9.12)**	**6.13 (3.11–12.09)**	**4.81 (2.14–10.84)**	**8.08 (4.35–15.01)**
Low	**1.92 (1.16–3.17)**	**1.83 (1.05–3.19)**	1.13 (0.53–2.39)	**1.82 (1.10–3.03)**
High	**2.24 (1.35–3.72)**	1.50 (0.83–2.72)	**2.23 (1.10–4.52)**	1.69 (0.99–2.87)

Note: Multinomial logistical regression, reference category = no exposure (*n* = 412), weighted results; significant values (*p* < 0.05) are in bold; OR = odds ratio; CI = confidence interval; SES household: very low = 0–1 on a score from 0 to 3; low = 1.33–1.67 on a score of 0–3; high = 2.00–2.33 on a score of 0–3; very high = 2.67–3.00 on a score of 0–3

### Associations between adverse childhood experiences and health risk behaviours

The explained variance of statistical models showed a range of 5–21% (Table 4). After controlling for sociodemographic factors, various risk factors for single types of HRBs were found; e.g., the adjusted odds ratio (AOR) of suicidality was 3.19 (95% CI: 1.96–5.19; *p* < 0.001) times higher for university students who were emotionally neglected than for those students who did not report emotional neglect. However, rather surprisingly, physical neglect had a significant effect on risky drinking (AOR = 0.31; 95% CI: 0.11–0.87; *p* = 0.026), as did emotional abuse (AOR = 0.44; 95% CI: 0.26–0.75; *p* = 0.003) and peer violence (AOR = 0.30; 95% CI: 0.11–0.80; *p* = 0.017) on drug abuse. Risk factors for multiple HRBs were experiencing emotional neglect or sexual abuse and growing up with mental illness in the household.

**Table 4:** Logistic and multinominal logistic regression analyses on associations between single types of adverse childhood experiences (ACEs) and single and multiple health risk behaviours (HRBs)

ACEs	Single types of HRBs						Multiple HRBs		
	Risky drinking (*n* = 299)	Smoking daily (*n* = 153)	Drug abuse (*n* = 206)	Early sexual intercourse (*n* = 279)	Multiple sexual partners (*n* = 63)	Suicidality (*n* = 138)	1 HRB (*n* = 418)	2 HRBs (*n* = 190)	3+ HRBs (*n* = 103)
	AOR (95% CI)	AOR (95% CI)	AOR (95% CI)	AOR (95% CI)	AOR (95% CI)	AOR (95% CI)	AOR (95% CI)	AOR (95% CI)	AOR (95% CI)
PA	1.21 (0.98–1.50)	0.35 (0.12–1.03)	0.96 (0.36–2.56)	1.17 (0.52–2.63)	1.72 (0.45–6.59)	1.81 (0.77–4.28)	1.20 (0.54–2.70)	1.19 (0.46–3.08)	0.42 (0.12–1.49)
EA	1.05 (0.63–1.76)	0.87 (0.49–1.53)	**0.44 (0.26–0.75)**	1.23 (0.75–2.02)	0.48 (0.20–1.14)	**1.92 (1.05–3.50)**	1.00 (0.63–1.61)	1.03 (0.57–1.86)	1.27 (0.64–2.54)
PN	**0.31 (0.11–0.87)**	0.96 (0.43–2.15)	0.46 (0.20–1.09)	0.55 (0.23–1.33)	0.75 (0.13–4.37)	0.90 (0.36–2.29)	1.17 (0.54–2.54)	0.57 (0.20–1.60)	0.70 (0.22–2.25)
EN	0.68 (0.41–1.11)	**1.78 (1.11–2.87)**	1.54 (1.0–2.37)	1.0 (0.64–1.57)	1.61 (0.77–3.37)	**3.19 (1.96–5.19)**	**1.57 (1.02–2.41)**	**1.90 (1.13–3.21)**	**1.96 (1.05–3.64)**
SA	0.96 (0.58–1.57)	0.91 (0.52–1.61)	**2.18 (1.37–3.47)**	**2.60 (1.69–4.01)**	1.34 (0.61–2.95)	1.03 (0.56–1.90)	1.30 (0.84–1.99)	**1.91 (1.15–3.17)**	**3.12 (1.77–5.48)**
SubA	1.31 (0.78–2.22)	**1.99 (1.15–3.44)**	**1.84 (1.11–3.03)**	1.10 (0.65–1.84)	0.44 (0.16–1.23)	0.94 (0.49–1.80)	1.11 (0.68–1.83)	1.35 (0.74–2.45)	1.10 (0.53–2.29)
MI	1.23 (0.86–1.74)	1.49 (0.98–2.27)	**1.67 (1.15–2.39)**	1.18 (0.82–1.70)	**2.09 (1.12–3.91)**	1.43 (0.90–2.27)	1.09 (0.79–1.50)	1.13 (0.74–1.73)	**2.12 (1.26–3.57)**
DV	1.08 (0.71–1.64)	1.02 (0.62–1.67)	**1.60 (1.06–2.42)**	1.34 (0.88–2.04)	**2.69 (1.35–5.34)**	1.16 (0.66–2.02)	1.30 (0.88–1.91)	1.52 (0.92–2.51)	1.60 (0.87–2.96)
SD	0.94 (0.64–1.38)	1.24 (0.79–1.96)	0.83 (0.55–1.25)	**1.60 (1.09–2.34)**	0.98 (0.49–1.98)	0.84 (0.49–1.43)	1.31 (0.93–1.84)	1.57 (1.0–2.45)	1.04 (0.57–1.90)
AD	1.20 (0.76–1.89)	1.55 (0.94–2.55)	**2.26 (1.45–3.51)**	1.12 (0.72–1.76)	1.43 (0.66–3.11)	1.62 (0.91–2.86)	1.32 (0.87–2.0)	1.56 (0.92–2.63)	1.68 (0.85–3.29)
FP	**1.95 (1.17–3.27)**	1.29 (0.73–2.27)	0.74 (0.42–1.30)	0.83 (0.48–1.44)	0.46 (0.16–1.31)	0.76 (0.40–1.43)	1.54 (0.95–2.51)	1.20 (0.63–2.27)	1.48 (0.73–3.02)
B	0.75 (0.49–1.15)	1.53 (0.98–2.39)	0.78 (0.50–1.20)	0.64 (0.41–1.01)	1.16 (0.57–2.37)	**2.31 (1.45–3.69)**	0.90 (0.61–1.33)	1.00 (0.60–1.66)	0.84 (0.45–1.57)
PV	1.59 (0.78–3.26)	1.32 (0.63–2.73)	**0.30 (0.11–0.80)**	**2.19 (1.11–4.29)**	1.02 (0.30–3.40)	0.77 (0.35–1.70)	1.16 (0.50–2.70)	1.40 (0.49–4.0)	2.39 (0.80–7.13)
*n*	1230	1228	1211	1199	1086	1199	1230	1230	1230
R^2^	0.050	0.114	0.141	0.101	0.083	0.210	0.118	0.118	0.118

Note: Logistical regression analysis for single types of HRBs; multinomial logistical regression analysis for multiple HRBs, reference category = no HRB (*n* = 755); all analyses adjusted for sociodemographic variables (gender, individual age, parental age, migration background, parental education, and family SES); weighted results; significant values (*p* < 0.05) are in bold; *AOR* adjusted odds ratio, *CI* confidence interval, *PA* physical abuse, *EA* emotional abuse, *PN* physical neglect, *EN* emotional neglect, *SA* sexual abuse, *SubA* substance abuse by household member, *MI* mental illness of household member, *DV* domestic violence, *SD* parental separation or divorce, *AD* parental absence or death, *FP* serious financial problems, *B* bullying, *PV* peer violence

**Fig. 1:**
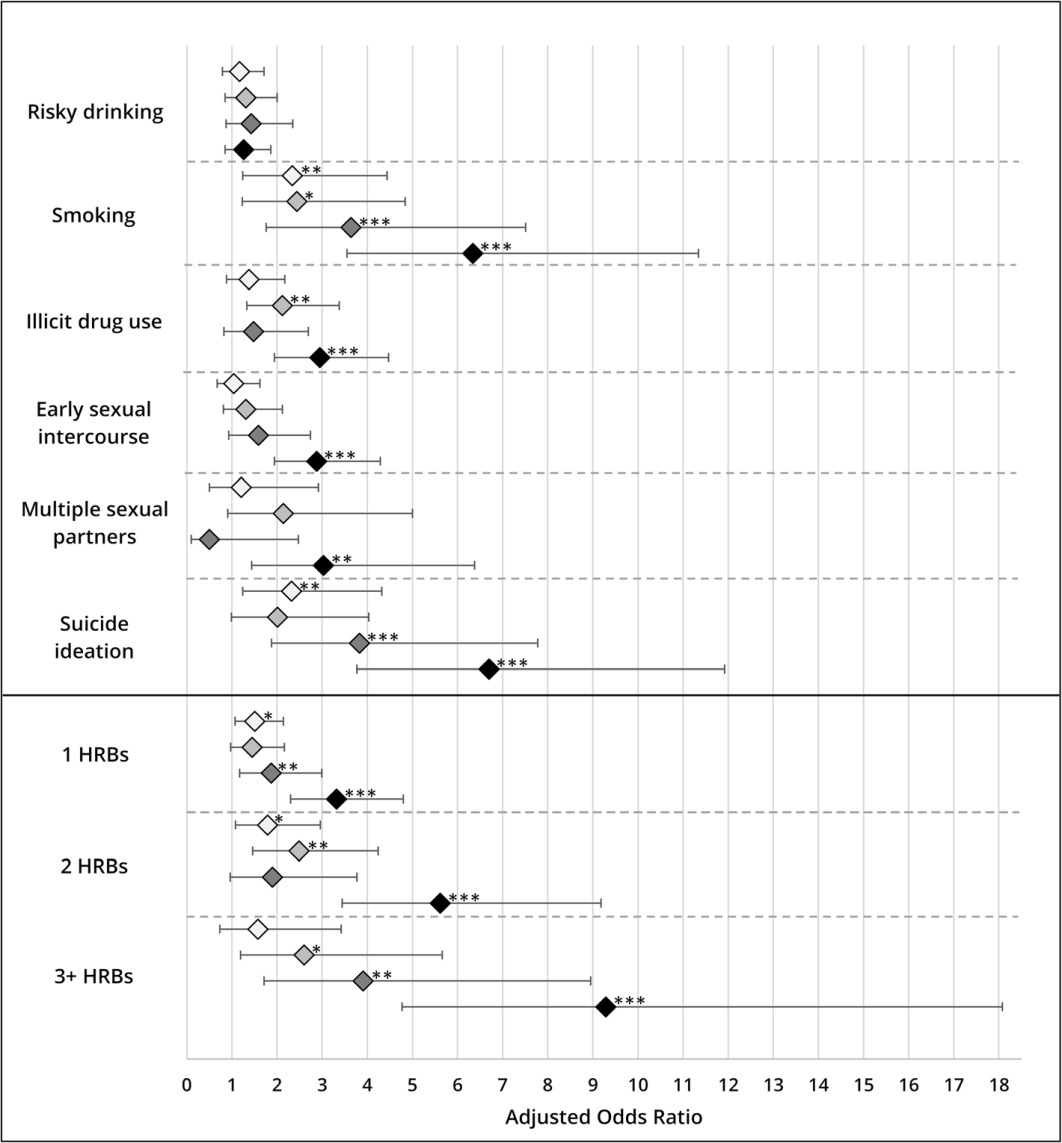
Associations between multiple types of adverse childhood experiences (ACEs) and single and multiple health risk behaviours (HRBs). Note: Logistical regression analysis for single types of HRBs (upper level); multinomial logistical regression analysis for multiple HRBs (lower level), reference category = no HRB (*n* = 755); all analyses adjusted for sociodemographic variables (gender, individual age, parental age, migration background, parental education, family SES); weighted results; *p < 0.05, **p < 0.01, ***p < 0.001

Figure 1 summarises the adjusted ORs for the relation between multiple ACEs and single as well as multiple HRBs. Experiencing four or more ACEs is associated with current smoking (AOR = 6.34; 95% CI: 3.55–11.34; *p* < 0.001), drug abuse (AOR = 2.95; 95% CI: 1.94–4.47; *p* < 0.001), early sexual intercourse (AOR = 2.88; 95% CI: 1.94–4.29; *p* < 0.001), multiple sexual partners (AOR = 3.03; 95% CI: 1.44–6.38; *p* = 0.004), suicidality (AOR = 6.70; 95% CI: 3.77–11.92; *p* < 0.001), one HRB (AOR = 3.32; 95% CI: 2.30–4.80; *p* < 0.001), two HRBs (AOR = 5.62; 95% CI: 3.44–9.18; *p* < 0.001), and three or more HRBs (AOR = 9.29; 95% CI: 4.77–18.08; *p* < 0.001). Some evidence of dose-response relationships was found for smoking, drug abuse, suicidality, and multiple HRBs.

## Discussion

This study was designed to determine the nature of ACEs and their possible associations with HRBs in adulthood. First, it was found that ACEs were highly prevalent among participating university students. Exposure to ACEs showed a range of 3.9 to 34.0%. Almost one quarter of the participants had a history of four or more ACEs, which, compared to the combined data provided by Bellis et al. [31], is three times higher than in Eastern European countries. Nevertheless, it must be acknowledged that this study investigated 13 types of ACEs, which is more than in most of the previous ACE studies – i.e. studies in the meta-analysis by Bellis et al. [31] measured 10 ACEs. On the one hand, measuring more ACEs may enhance the ecological and cross-cultural validity [48], but on the other hand, this is at the expense of reduced comparability of data. Second, we sought to determine the sociodemographic factors affecting the occurrence of single and multiple ACEs. Since no single factor was found as the main explanation, it must be assumed that the mechanism between sociodemographic factors and a child’s exposure to ACEs is multifactorial. However, the study revealed that a high SES in childhood may protect against the occurrence of almost all dimensions of single as well as multiple ACEs. These findings agree with a meta-analysis by Stith et al. [60] and other publications [61, 62], which demonstrated that exposure to child maltreatment is inversely associated with household SES. Third, statistical analyses revealed many ACEs which might have altered the college students’ health behaviour; e.g., growing up with mental illness in the household can be associated with drug abuse. This finding is supported by earlier European studies [61, 63–67]. As postulated by Felitti et al. [1], dose-response relationships between the number of ACEs and HRBs were found. In accordance with previous international studies [6, 31, 68], a definite graded dose-response relationship was found for smoking. Apart from risky drinking, all statistical models showed that exposure to four or more ACEs is linked with the adoption of smoking, drug abuse, early sexual intercourse, multiple sexual partners, and suicidality. This agrees with previous studies [12]. Finally, it was found that the number of ACEs also determines the number of HRBs. Graded dose-response relationships were found for one, two and three or more HRBs.

### Limitations

A major limitation of this investigation is the lack of causal inferences due to the cross-sectional design. Even though Springer et al. [69] argue that “many of the criteria for a causal relationship are met” in retrospective ACE studies, we agree with previous critical authors [70–72] that a causal relationship between ACEs and negative health outcomes in adulthood cannot be established on the basis of cross-sectional data. Another major drawback is the fact that the analyses rely on self-reported data (e.g., risk of socially desirable answers). Moreover, the low and unknown response rates of faculties and students may have caused a sampling bias (non-representative sample). For example, it was observed that more women than men took part in the study, which was then retrospectively adjusted using a weighting factor. Although generalisability does not necessarily require representativeness [73, 74], the results of this study can only be transferred to the total population of all German public university students to a limited extent. A further source of error could be the retrospective survey design. When asking adults about events during their childhood, it is likely that the collected data may be flawed by recall bias; e.g., participants might not remember the events because they were too young, they did not realise at the time that a certain kind of behaviour is abusive [72], or they simply forgot as a result of traumatic experiences [75, 76]. Thus, retrospective studies might underestimate the prevalence rates of ACEs, which again raises the possibility that the true effects of ACEs on HRBs might be even greater. Another potential bias could be that college students typically underreport their level of HRBs [77, 78]. Again, this may conceal the true effect size of ACEs. Otherwise, it was also found that emotional abuse and peer violence decreased the likelihood of drug abuse in adulthood. A possible explanation for this is that those children who have suffered from emotional abuse or peer violence are less socially active [79], and thus have only limited access to peer groups in which they may consume illegal drugs [80, 81].

### Practical implications

Notwithstanding the limitations outlined above, this analysis increases knowledge about ACEs in Germany by applying a non-clinical sample. This is one of the first studies to assess multiple HRBs as an outcome variable [28]. As a result, the findings have a number of important implications for future policy and practice.

A major implication is that these results can be supportive for public health officials to increase awareness about the scope and negative impact of ACEs. For example, this evidence might persuade political decision-makers to set priorities, allocate resources and approve legal child protection policies [82, 83]. A viable argument could be to stress the cumulative negative effects of ACEs on smoking behaviour. This would be of particular interest for decision-makers because tobacco use is known to alter both individual health [84] and overall societal healthcare costs [85].

Furthermore, paediatricians and other healthcare workers who are aware of the effects of ACEs can intervene during the early stages of life. This could mean pointing out the long-term effects of ACEs to caregivers and thus sensitise parents to their own and others’ parenting behaviour. Further groups that might benefit are psychologists, psychiatrists and social workers [86]. Assessing the history of potential ACEs among their clients offers a unique opportunity to explain the pathways of stress, depressive symptoms and suicidal behaviour. In the psychotherapy setting, patients can understand how their earlier experiences (e.g., emotional neglect or bullying) could influence their current behaviour (e.g., suicidality), which might be supportive in mobilising coping strategies and recovery [87].

Although it seems advisable to broaden the scope of screening for ACEs, Finkelhor [88] calls for prudence. He argues that, before implementing large-scale screening programmes, three key challenges need to be addressed: (1) develop effective interventions, (2) determine possible adverse effects or costs of screening (stigma, psychological stress, financial costs, overtreatment, etc.), and (3) define standardised screening tools. Only then will routine screenings in different German settings – such as medical settings [89–91], schools [92], home visiting programmes [93], or even prisons [94] – be feasible to implement, and serve the public interest.

The present dataset is part of a growing body of literature, and can help inform practitioners in healthcare [95], social care [86], or in the educational sector [96]. This could provide the starting point for designing and implementing targeted interventions to decrease the burden of ACEs, such as the finding that health professionals aiming to provide home visiting services for families should prioritise low-income families [97, 98], and provide culturally sensitive, low-threshold information on positive parenting [99].

In summary, public health efforts should shift their focus to preventing ACEs before they occur; to designing, testing, and implementing interventions to increase capacity among children and adults; and to strengthening regulatory policies to ensure a healthy environment throughout the life-cycle, as suggested by Hughes et al. [12].

## Conclusions

These findings confirm that ACEs are fairly common among German public university students, and so can be used to predict the occurrence of HRBs in adulthood. The most obvious finding was that experiencing multiple stressful events during childhood can increase the likelihood of performing single and multiple health-harming behaviours throughout the life course. Thus, reducing the number of ACEs should decrease HRBs, which account for many of the leading causes of morbidity and death.

These findings have far-reaching consequences for public health professionals and researchers and should therefore be acknowledged and addressed accordingly. Scientists in various fields need to explore and optimise screening methods, prevention programmes and therapeutic interventions. New insights from the neurosciences help us to better understand the long-term consequences of traumatic experiences during childhood. Developing a global network of researchers aiming to share knowledge about ACEs and their effects, and sharing best practice experiences on public health interventions, is a key component for future progress.

Child advocates bear a great responsibility: They must speak up for those who cannot speak for themselves. In order to avoid further suffering of children, and the accordingly higher risk of HRBs, decision-makers in Germany need to start thinking about ACEs and take action to reduce this burden. A potential role model for the implementation of such trauma-informed approaches could be the community capacity initiative of the Family Policy Council in Washington State/USA [100]. In two studies, the programme deliverers have proven that building community capacity (support and resources within communities) can have positive effects on reducing child and family problems, as well as on reducing ACE prevalence rates [100].

## Additional file

Additional file 1: Measurements and responses to different types of adverse childhood experiences. (DOCX 21 kb)

## Abbreviations

ACE: Adverse childhood experience
ACE-IQ: Adverse Childhood Experiences International Questionnaire
AD: Parental absence or death
AOR: Adjusted odds ratios
AUDIT-C: Alcohol Use Disorders Identification Test
B: Bullying
CASI: Computer-assisted self-interview
CI: Confidence interval
DV: Domestic violence
EA: Emotional abuse
EN: Emotional neglect
FP: Serious financial problems
HRB: Health risk behaviour
MI: Mental illness in the household
OR: Odds ratio
PA: Physical abuse
PN: Physical neglect
PV: Peer violence
RKI: Robert Koch-Institute
RQ: Research question
SA: Sexual abuse
SD: Parental separation or divorce
SES: Socio-economic status
SubA: Substance abuse by household member
VIF: Variance inflation factor
WHO: World Health Organization

## References

[1] Felitti VJ, Anda RF, Nordenberg D, Williamson DF, Spitz AM, Edwards V (1998). Relationship of childhood abuse and household dysfunction to many of the leading causes of death in adults: the adverse childhood experiences (ACE) study. Am J Prev Med.

[2] Anda RF, Dong M, Brown DW, Felitti VJ, Giles WH, Perry GS (2009). The relationship of adverse childhood experiences to a history of premature death of family members. BMC Public Health.

[3] Brown DW, Anda R, Tiemeier H, Felitti VJ, Edwards VJ, Croft JB, Giles WH (2009). Adverse childhood experiences and the risk of premature mortality. Am J Prev Med.

[4] Tröbs RB, González-Vásquez R, Barenberg K (2010). Kindesmisshandlung – nicht unfallbedingte Verletzungen bei Kindern. OP-Journal.

[5] Douglas EM (2017). Child maltreatment fatalities in the United States. Four decades of policy, program, and professional responses.

[6] Brown DW, Anda RF, Felitti VJ, Edwards VJ, Malarcher AM, Croft JB, Giles WH (2010). Adverse childhood experiences are associated with the risk of lung cancer: a prospective cohort study. BMC Public Health.

[7] Anda RF, Brown DW, Dube SR, Bremner JD, Felitti VJ, Giles WH (2008). Adverse childhood experiences and chronic obstructive pulmonary disease in adults. Am J Prev Med.

[8] Dong M, Giles WH, Felitti VJ, Dube SR, Williams JE, Chapman DP, Anda RF (2004). Insights into causal pathways for ischemic heart disease. Adverse Childhood Experiences Study Circ.

[9] Kessler RC, McLaughlin KA, Green JG, Gruber MJ, Sampson NA, Zaslavsky AM (2010). Childhood adversities and adult psychopathology in the WHO world mental health surveys. Br J Psychiatry.

[10] Chapman DP, Whitfield CL, Felitti VJ, Dube SR, Edwards VJ, Anda RF (2004). Adverse childhood experiences and the risk of depressive disorders in adulthood. J Affect Disord.

[11] Edwards VJ, Holden GW, Felitti VJ, Anda RF (2003). Relationship between multiple forms of childhood maltreatment and adult mental health in community respondents: results from the adverse childhood experiences study. Am J Psychiatry.

[12] Hughes K, Bellis MA, Hardcastle KA, Sethi D, Butchart A, Mikton C (2017). The effect of multiple adverse childhood experiences on health: a systematic review and meta-analysis. Lancet Public Health.

[13] Dube SR, Miller JW, Brown DW, Giles WH, Felitti VJ, Dong M, Anda RF (2006). Adverse childhood experiences and the association with ever using alcohol and initiating alcohol use during adolescence. J Adolesc Health.

[14] Dube SR, Anda RF, Felitti VJ, Edwards VJ, Croft JB (2002). Adverse childhood experiences and personal alcohol abuse as an adult. Addict Behav.

[15] Forster M, Grigsby TJ, Rogers CJ, Benjamin SM (2018). The relationship between family-based adverse childhood experiences and substance use behaviors among a diverse sample of college students. Addict Behav.

[16] Anda RF, Croft JB, Felitti VJ, Nordenberg D, Giles WH, Williamson DF, Giovino GA (1999). Adverse childhood experiences and smoking during adolescence and adulthood. JAMA.

[17] Chung EK, Nurmohamed L, Mathew L, Elo IT, Coyne JC, Culhane JF (2010). Risky health behaviors among mothers-to-be: the impact of adverse childhood experiences. Acad Pediatr.

[18] Dube SR, Felitti VJ, Dong M, Chapman DP, Giles WH, Anda RF (2003). Childhood abuse, neglect, and household dysfunction and the risk of illicit drug use: the adverse childhood experiences study. Pediatrics.

[19] Hillis SD, Anda RF, Felitti VJ, Marchbanks PA (2001). Adverse childhood experiences and sexual risk behaviors in women: a retrospective cohort study. Fam Plan Perspect.

[20] Dietz PM, Spitz AM, Anda RF, Williamson DF, McMahon PM, Santelli JS (1999). Unintended pregnancy among adult women exposed to abuse or household dysfunction during their childhood. JAMA.

[21] Hahm HC, Lee Y, Ozonoff A, Wert MJ (2010). The impact of multiple types of child maltreatment on subsequent risk behaviors among women during the transition from adolescence to young adulthood. J Youth Adolesc.

[22] Dube SR, Anda RF, Felitti VJ, Chapman DP, Williamson DF, Giles WH (2001). Childhood abuse, household dysfunction, and the risk of attempted suicide throughout the life span: findings from the adverse childhood experiences study. JAMA.

[23] Scott BG, Burke NJ, Weems CF, Hellman JL, Carrión VG (2013). The interrelation of adverse childhood experiences within an at-risk pediatric sample. J Child Adolesc Trauma.

[24] Dong M, Anda RF, Felitti VJ, Dube SR, Williamson DF, Thompson TJ (2004). The interrelatedness of multiple forms of childhood abuse, neglect, and household dysfunction. Child Abuse Negl.

[25] Finkelhor D, Ormrod RK, Turner HA (2007). Poly-victimization: a neglected component in child victimization. Child Abuse Negl.

[26] Bronfenbrenner U (1977). Toward an experimental ecology of human development. Am Psychol.

[27] Krug EG, Dahlberg LL, Mercy JA, Zwi AB, Lozano R (2002). World report on violence and health.

[28] Bellis MA, Hardcastle K, Ford K, Hughes K, Ashton K, Quigg Z, Butler N (2017). Does continuous trusted adult support in childhood impart life-course resilience against adverse childhood experiences-a retrospective study on adult health-harming behaviours and mental well-being. BMC Psychiatry.

[29] Stoltenborgh M, Bakermans-Kranenburg MJ, Alink LR, IJzendoorn MH (2015). The prevalence of child maltreatment across the globe: review of a series of meta-analyses. Child Abuse Rev.

[30] Sethi D, Bellis M, Hughes K, Gilbert R, Mitis F, Galea G (2013). European report on preventing child maltreatment.

[31] Bellis MA, Hughes K, Leckenby N, Jones L, Baban A, Kachaeva M (2014). Adverse childhood experiences and associations with health-harming behaviours in young adults: surveys in eight eastern European countries. Bull World Health Organ.

[32] Kaess M, Parzer P, Mattern M, Plener PL, Bifulco A, Resch F, Brunner R (2013). Adverse childhood experiences and their impact on frequency, severity, and the individual function of nonsuicidal self-injury in youth. Psychiatry Res.

[33] Hardt J, Vellaisamy P, Schoon I (2010). Sequelae of prospective versus retrospective reports of adverse childhood experiences. Psychol Rep.

[34] Wingenfeld K, Schäfer I, Terfehr K, Grabski H, Driessen M, Grabe H, Spitzer C (2011). Reliable, valide und ökonomische Erfassung früher Traumatisierung: Erste psychometrische Charakterisierung der deutschen Version des Adverse Childhood Experiences Questionnaire (ACE). PPmP.

[35] Schulz A, Schmidt CO, Appel K, Mahler J, Spitzer C, Wingenfeld K (2014). Psychometric functioning, sociodemographic variability of childhood maltreatment in the general population and its effects of depression. Int J Methods Psychiatr Res.

[36] Glaesmer H, Schulz A, Häuser W, Freyberger HJ, Brähler E, Grabe HJ (2013). Der Childhood Trauma Screener (CTS)–Entwicklung und Validierung von Schwellenwerten zur Klassifikation. Psychiatr Prax.

[37] Iffland B, Brähler E, Neuner F, Häuser W, Glaesmer H (2013). Frequency of child maltreatment in a representative sample of the German population. BMC Public Health.

[38] Appel K, Schwahn C, Mahler J, Schulz A, Spitzer C, Fenske K (2011). Moderation of adult depression by a polymorphism in the FKBP5 gene and childhood physical abuse in the general population. Neuropsychopharmacology.

[39] Häuser W, Schmutzer G, Brähler E, Glaesmer H. Misshandlungen in Kindheit und Jugend. Dtsch Arztebl. 2011;108(17):287–94. 10.3238/arztebl.2011.0287 PMC3103979 21629512

[40] Wetzels P (1997). Zur Epidemiologie physischer und sexueller Gewalterfahrungen in der Kindheit.

[41] World Health Organization (WHO) (1999). Report of the Consultation on Child Abuse Prevention.

[42] Statistisches Bundesamt: Studierende: Deutschland, Semester, Nationalität, Geschlecht, Hochschulen. https://www-genesis.destatis.de/genesis/online/logon?sequenz=tabelleErgebnis&selectionname=21311-0002 (2017). Accessed 09 Aug 2017.

[43] Bundesinstitut für Bevölkerungsforschung: Durchschnittsalter der Bevölkerung. http://www.bib-demografie.de/SharedDocs/Glossareintraege/DE/D/durchschnittsalter_bevoelkerung.html Accessed 22 Nov 2017.

[44] Statistisches Bundesamt (2017). Bevölkerung und Erwerbstätigkeit. Bevölkerung mit Migrationshintergrund.

[45] Cohen S (2010). SC childhood interview.

[46] John-Henderson NA, Marsland AL, Kamarck TW, Muldoon MF, Manuck SB (2016). Childhood socioeconomic status and the occurrence of recent negative life events as predictors of circulating and stimulated levels of interleukin-6. Psychosom Med.

[47] World Health Organization (WHO): Adverse Childhood Experiences International Questionnaire (ACE-IQ). http://www.who.int/violence_injury_prevention/violence/activities/adverse_childhood_experiences/en/ (2017). Accessed 13 July 2017.

[48] Mersky JP, Janczewski CE, Topitzes J (2016). Rethinking the measurement of adversity: moving toward second-generation research on adverse childhood experiences. Child Maltreat.

[49] Bush K, Kivlahan DR, McDonell MB, Fihn SD, Bradley KA (1998). The AUDIT alcohol consumption questions (AUDIT-C): an effective brief screening test for problem drinking. Arch Intern Med.

[50] Robert Koch-Institut (RKI) (2014). Beiträge zur Gesundheitsberichterstattung des Bundes. Daten und Fakten: Ergebnisse der Studie “Gesundheit in Deutschland aktuell 2012”.

[51] DeMartini KS, Carey KB (2012). Optimizing the use of the AUDIT for alcohol screening in college students. Psychol Assess.

[52] Robert Koch-Institut (RKI): DEGS: Studie zur Gesundheit Erwachsener in Deutschland. http://www.rki.de/DE/Content/Gesundheitsmonitoring/Studien/Degs/degs_node.html (2017). Accessed 07 Aug 2017.

[53] Gawlik KS, Melnyk BM, Tan A (2017). An epidemiological study of population health reveals social smoking as a major cardiovascular risk factor. Am J Health Promot.

[54] Bundesministerium für Gesundheit (2016). Drogen- und Suchtbericht.

[55] Orth B (2016). Die Drogenaffinität Jugendlicher in der Bundesrepublik Deutschland 2015. Rauchen, Alkoholkonsum und Konsum illegaler Drogern: aktuelle Verbreitung und Trends. BZgA-Forschungsbericht.

[56] White Hughto JM, Biello KB, Reisner SL, Perez-Brumer A, Heflin KJ, Mimiaga MJ (2016). Health risk behaviors in a representative sample of bisexual and heterosexual female high school students in Massachusetts. J Sch Health.

[57] Kuortti M, Kosunen E (2009). Risk-taking behaviour is more frequent in teenage girls with multiple sexual partners. Scand J Prim Health Care.

[58] Ethier KA, Kershaw T, Niccolai L, Lewis JB, Ickovics JR (2003). Adolescent women underestimate their susceptibility to sexually transmitted infections. Sex Transm Infect.

[59] World Health Organization (WHO): Global School-Based Student Health Survey (GSHS). 2013 Core Questionnaire Modules. http://www.who.int/chp/gshs/methodology/en/ (2013). Accessed 10 July 2017.

[60] Stith SM, Liu T, Davies LC, Boykin EL, Alder MC, Harris JM (2009). Risk factors in child maltreatment: a meta-analytic review of the literature. Aggress Violent Behav.

[61] Qirjako G, Burazeri G, Sethi D, Miho V (2013). Community survey on prevalence of adverse childhood experiences in Albania.

[62] Garbarino J, Kostelny K (1992). Child maltreatment as a community problem. Child Abuse Negl.

[63] Paunović M, Marković M, Vojvodić K, Nešković A, Sethi D, Grbić M (2015). Survey of adverse childhood experiences among Serbian university students. Report from the 2013/2014 Survey.

[64] Kachaeva MA, Sethi D, Badmaeva VD, Novozhilov AV, Ivanov AV (2014). Survey on the prevalence of adverse childhood experiences among young people in the Russian Federation.

[65] Institute of Public Health Montenegro (2013). Survey on adverse childhood experiences in Montenegro.

[66] Ulukol B, Kahiloğulları AK, Sethi D (2014). Adverse childhood experiences survey among university students in Turkey.

[67] Baban A, Cosma A, Balázsi R, Sethi D, Olsavszky V. Survey of adverse childhood experiences among Romanian university students. Study Report from the 2012 Survey. Copenhagen: WHO Regional Office for Europe; 2013.

[68] Edwards VJ, Anda RF, Gu D, Dube SR, Felitti VJ (2007). Adverse childhood experiences and smoking persistence in adults with smoking-related symptoms and illness. Perm J.

[69] Springer KW, Sheridan J, Kuo D, Carnes M (2003). The long-term health outcomes of childhood abuse. J Gen Intern Med.

[70] Sareen J, Fleisher W, Cox BJ, Hassard S, Stein MB (2005). Childhood adversity and perceived need for mental health care: findings from a Canadian community sample. J Nerv Ment Dis.

[71] Raphael KG, Chandler HK, Ciccone DS (2004). Is childhood abuse a risk factor for chronic pain in adulthood?. Curr Pain Headache Rep.

[72] Hardt J, Rutter M (2004). Validity of adult retrospective reports of adverse childhood experiences: review of the evidence. J Child Psychol Psychiatry.

[73] Rothman KJ, Gallacher JE, Hatch EE (2013). Why representativeness should be avoided. Int J Epidemiol.

[74] Richiardi L, Pizzi C, Pearce N (2013). Commentary: representativeness is usually not necessary and often should be avoided. Int J Epidemiol.

[75] Edwards VJ, Fivush R, Anda RF, Felitti VJ, Nordenberg DF (2001). Autobiographical memory disturbances in childhood abuse survivors. J Aggress Maltreat Trauma.

[76] Chu JA, Frey LM, Ganzel BL, Matthews JA (1999). Memories of childhood abuse: dissociation, amnesia, and corroboration. Am J Psychiatry.

[77] White AM, Kraus CL, McCracken LA, Swartzwelder HS (2003). Do College students drink more than they think? Use of a free-pour paradigm to determine how college students define standard drinks. Alcohol Clin Exp Res.

[78] White AM, Kraus CL, Flom JD, Kestenbaum LA, Mitchell JR, Shah K, Swartzwelder HS (2005). College students lack knowledge of standard drink volumes: implications for definitions of risky drinking based on survey data. Alcohol Clin Exp Res.

[79] Al Odhayani A, Watson WJ, Watson L (2013). Behavioural consequences of child abuse. Can Fam Physician.

[80] Bryant AL, Schulenberg JE, O'malley PM, Bachman JG, Johnston LD (2003). How academic achievement, attitudes, and behaviors relate to the course of substance use during adolescence: a 6-year, multiwave national longitudinal study. J Res Adolesc.

[81] Svensson R (2000). Risk factors for different dimensions of adolescent drug use. J Child Adolesc Subst Abuse.

[82] Anda RF, Brown DW (2007). Root causes and organic budgeting: funding health from conception to the grave. Ped Health.

[83] Snyder H, Oshiro C (2006). Persuading decision makers to act for better health.

[84] Neubauer S, Welte R, Beiche A, Koenig HH, Buesch K, Leidl R (2006). Mortality, morbidity and costs attributable to smoking in Germany: update and a 10-year comparison. Tob Control.

[85] Ruff LK, Volmer T, Nowak D, Meyer A (2000). The economic impact of smoking in Germany. Eur Respir J.

[86] Larkin H, Felitti VJ, Anda RF (2014). Social work and adverse childhood experiences research – implications for practice and health policiy. Soc Work Public Health.

[87] Jacob KS (2015). Recovery model of mental illness: a complementary approach to psychiatric care. Indian J Psychol Med.

[88] Finkelhor D. Screening for adverse childhood experiences (ACEs): cautions and suggestions. Child Abuse Negl. 2017. 10.1016/j.chiabu.2017.07.016. 10.1016/j.chiabu.2017.07.016 28784309

[89] Shafer MB. Nurse Practitioner Screening for Adverse Childhood Outcomes in Adult Primary Care. https://scholarworks.umass.edu/nursing_dnp_capstone/107/ (2018). Accessed 22 May 2018.

[90] Glowa PT, Olson AL, Johnson DJ (2016). Screening for adverse childhood experiences in a family medicine setting: a feasibility study. J Am Board Fam Med.

[91] Purewal SK, Bucci M, Gutiérrez Wang L, Koita K, Silvério Marques S, Oh D, Burke HN (2016). Screening for adverse childhood experiences (ACEs) in an integrated pediatric care model. Zero to Three.

[92] Eklund K, Rossen E (2016). Guidance for trauma screening in schools.

[93] Johnson K, Woodward A, Swenson S, Weis C, Gunderson M, Deling M, Cristiani V, Lynch B (2017). Parents' adverse childhood experiences and mental health screening using home visiting programs: a pilot study. Public Health Nurs.

[94] Wolff N, Shi J (2012). Childhood and adult trauma experiences of incarcerated persons and their relationship to adult behavioral health problems and treatment. Int J Environ Res Public Health.

[95] Oral R, Ramirez M, Coohey C, Nakada S, Walz A, Kuntz A, Benoit J, Peek-Asa C (2015). Adverse childhood experiences and trauma informed care: the future of health care. Pediatr Res.

[96] Walkley M, Cox TL (2013). Building trauma-informed schools and communities. Children Schools.

[97] Levey EJ, Gelaye B, Bain P, Rondon MB, Borba CP, Henderson DC, Williams MA (2017). A systematic review of randomized controlled trials of interventions designed to decrease child abuse in high-risk families. Child Abuse Negl.

[98] Bilukha O, Hahn RA, Crosby A, Fullilove MT, Liberman A, Moscicki E (2005). The effectiveness of early childhood home visitation in preventing violence – a systematic review. Am J Prev Med.

[99] Neuhauser A, Ramseier E, Schaub S, Burkhardt SC, Templer F, Lanfranchi A (2015). Hard to reach families – a methodological approach to early recognition, recruitment, and randomization in an intervention study. Ment Health Prev.

[100] Hall J, Porter L, Longhi D, Becker-Green J, Dreyfus S (2012). Reducing adverse childhood experiences (ACE) by building community capacity: a summary of Washington family policy council research findings. J Prev Interv Community.

