# Supplementary material for: How adverse childhood experiences relate to single and multiple health risk behaviours in German public university students: a cross-sectional analysis
Source: BMC Public Health. 2018 Aug 13;18:1005. doi: 10.1186/s12889-018-5926-3 (PMC6090638; doi:10.1186/s12889-018-5926-3)
Supplement: Supplementary file 1 — Measurements and responses to different types of adverse childhood experiences. (DOCX 21 kb) [file 12889_2018_5926_MOESM1_ESM.docx]

**Additional file 1**

**Measurements and responses to different types of adverse childhood experiences**

| ***During the first 18 years of your life…*** | **Response ^a^** | **Prevalence** | |
| --- | --- | --- | --- |
|  |  | ***n* ^b^** | **% ^c^** |
| **Physical abuse** |  | 62 | 3.9 |
| 1. *…how often did a parent, guardian or household member punch, kick or beat you?* | never, once, a few times, **many times** | 60 | 3.8 |
| 1. *…how often did a parent, guardian or household member hit you with an object?* | never, once, a few times, **many times** | 24 | 1.6 |
| **Emotional abuse** |  | 300 | 19.6 |
| 1. *…how often did a parent, guardian, or household member humiliate you?* | never, once, a few times, **many times** | 135 | 8.2 |
| 1. *…how often did a parent, guardian, or household member yell, scream or swear at you, or insult you?* | never, once, a few times, **many times** | 283 | 18.2 |
| 1. *…how often did a parent, guardian, or household member seriously threaten to, or actually, throw you out of the house?* | never, once, a few times, **many times** | 49 | 3.3 |
| **Physical neglect** |  | 64 | 4.6 |
| 1. *…how often did your parents/ guardians not give you enough food even when they could easily have done so?* | never, once, a few times, **many times** | 11 | 0.7 |
| 1. *…how often was at least one parent too drunk or intoxicated by drugs (e.g., amphetamine, LSD or marijuana) to take care of you when you have needed them?* | never, once, a few times, **many times** | 50 | 3.8 |
| 1. *…how often did your parents/ guardians not send you to school even when it was available?* | never, once, a few times, **many times** | 8 | 0.5 |
| **Emotional neglect** |  | 266 | 19.1 |
| 1. *…how often did your parents/ guardians understand your problems and worries?* | **never**, **rarely**, sometimes, most of the time, always | 213 | 14.9 |
| 1. *…how often did your parents/ guardians really know what you were doing with your free time when you were not at school or work?* | **never**, **rarely**, sometimes, most of the time, always | 123 | 9.4 |

| ***During the first 18 years of your life…*** | **Response ^a^** | **Prevalence** | |
| --- | --- | --- | --- |
|  |  | ***n* ^b^** | **% ^c^** |
| **Sexual abuse** |  | 215 | 12.3 |
| 1. *…how often did someone touch or fondle you in a sexual way when you did not want them to?* | never, **once**, **a few times**, **many times** | 184 | 10.3 |
| 1. *…how often did someone make you touch their body in a sexual way when you did not want them to?* | never, **once**, **a few times**, **many times** | 79 | 4.5 |
| 1. *…how often did someone attempt oral, anal, or vaginal intercourse with you when you did not want them to?* | never, **once**, **a few times**, **many times** | 99 | 5.7 |
| 1. *…how often did someone actually have oral, anal, or vaginal intercourse with you when you did not want them to?* | never, **once**, **a few times**, **many times** | 61 | 3.4 |
| **Substance abuse by a household member** |  | 184 | 12.5 |
| 1. *...did you live with a household member who was an alcoholic, or misused illegal or prescription drugs?* | no, **yes** | 184 | 12.5 |
| **Mental illness of a household member** |  | 453 | 32.1 |
| 1. *...did you live with a household member who was depressed, mentally ill or suicidal?* | no, **yes** | 453 | 32.1 |
| **Domestic violence** |  | 473 | 34.0 |
| 1. *…did you see or hear a household member in your home being humiliated?* | never, once, a few times, **many times** | 187 | 11.4 |
| 1. *…did you see or hear a household member in your home being yelled at, screamed at, sworn at, or insulted?* | never, once, a few times, **many times** | 376 | 26.0 |
| 1. *…did you see or hear a household member in your home being punched, kicked, or beaten up?* | never, once, **a few times**, **many times** | 255 | 18.1 |
| 1. *…did you see or hear a household member in your home being hit with an object?* | never, once, **a few times**, **many times** | 150 | 10.6 |
| **Parental separation/divorce** |  | 408 | 28.1 |
| 1. *…were your parents ever separated or divorced?* | no, **yes** | 408 | 28.1 |

| ***During the first 18 years of your life…*** | **Response ^a^** | **Prevalence** | |
| --- | --- | --- | --- |
|  |  | ***n* ^b^** | **% ^c^** |
| **Absence of caregiver** |  | 230 | 16.6 |
| 1. *…was your mother, your father or guardian absent from your life for a long time?* | no, **yes** | 230 | 16.6 |
| **Death of caregiver** |  | 71 | 4.3 |
| 1. *…did your mother, your father or guardian die?* | no, **yes** | 71 | 4.3 |
| **Financial problems** |  | 150 | 9.8 |
| 1. *…how often did your family experience serious financial problems?* | never, rarely, sometimes, **most of the time**, **always** | 150 | 9.8 |
| **Bullying** |  | 251 | 20.8 |
| 1. *…how often were you bullied?* | never, a few times, **many times** | 251 | 20.8 |
| **Physical Fighting** |  |  | 4.6 |
| 1. *…how often were you in a physical fight with other children or adolescents?* | never, once, a few times, **many times** | 40 | 4.6 |
| **Violence in the community ^d^** |  | 15 | 1.8 |
| 1. *…how often did you see or hear someone being beaten up in real life?* | never, once, a few times, **many times** | 14 | 1.7 |
| 1. *…how often did you see or hear someone being threatened with a knife or gun in real life?* | never, once, a few times, **many times** | - | - |
| 1. *…how often did you see or hear someone being stabbed or shot in real life?* | never, once, a few times, **many times** | - | - |

Note: Failures to reply (“can’t/don’t want to say”) were treated as missing values; *N*= 1,466 German college students.

^a^ Answers were dichotomized: bold responses indicate that persons replying in such way were marked as positive. If at least one question to each type of adverse childhood experiences is positive, the person was treated as having been exposed to the respective traumatic event during childhood.

^b^ Results on absolute frequencies (*n*) are unweighted.

^c^ Results on relative frequencies (%) are weighted.

^d^ Due to accuracy issues of the data, small numbers (n<5) are being suppressed.
